# Manganese Phosphatizing Coatings: The Effects of Preparation Conditions on Surface Properties

**DOI:** 10.3390/ma11122585

**Published:** 2018-12-18

**Authors:** Jakub Duszczyk, Katarzyna Siuzdak, Tomasz Klimczuk, Judyta Strychalska-Nowak, Adriana Zaleska-Medynska

**Affiliations:** 1 Department of Environmental Technology, Faculty of Chemistry, University of Gdansk, Wita Stwosza str. 63, 80-308 Gdansk, Poland; 2 Mayr, Rojów, Hetmanska str. 1, 63-500 Ostrzeszow, Poland; 3 The Szewalski Institute of Fluid-Flow Machinery Polish Academy of Sciences, Fiszera str. 14, 80-231 Gdansk, Poland; ksiuzdak@imp.gda.pl; 4 Faculty of Applied Physics and Mathematics, Gdansk University of Technology, G. Narutowicza str. 11-12, 80-233 Gdansk, Poland; tomasz.klimczuk@pg.edu.pl (T.K.); judyta.strychalska@gmail.com (J.S.-N.)

**Keywords:** phosphating, manganese phosphating coatings, surface protection, conversion coatings

## Abstract

Manganese phosphate coating could be used to protect the surface of steel products. However, it is essential to determine the effects which process parameters, as well as the types of additives used, have on the efficiency of coating deposition. Thus, we present here a process of phosphatization of low-alloy steel (for 15 min at 95 °C) in manganese/nickel baths followed by a passivation process with the use of a silicon and zircon compounds. The microstructure and morphology of the surface were analyzed by SEM EDX and XRD methods. The obtained results showed that the manganese phosphate could be effectively formed at 95 °C in the solution containing nickel and guanidine derivatives. Anodic polarization of manganese coating was investigated in 0.5 M KCl by the analysis of polarization resistance. The effects of the activation process on corrosion properties of the coating have been examined. It was observed that an increased concentration of activating substances in the activation bath results in the enhancement of corrosion resistance.

## 1. Introduction

Phosphate coatings are the most often used form of steel surface treatment due to the reasons which include their good adhesion, high corrosion resistance, improved abrasive resistance of the structure, and acceptable costs of the manufacturing [1,2,3,4]. Corrosion protection for steel could be achieved by the application of modified phosphate [2,3,4], zinc phosphate [5,6,7,8,9,10,11,12,13,14,15,16,17,18], iron phosphate [19,20,21], as well as manganese phosphate coatings [22,23,24,25,26,27,28,29,30]. It was found that, of the above-mentioned phosphate coatings, the manganese phosphate coatings have the highest hardness in addition to their remarkable corrosion and wear resistance [30]. Despite the existence of some patents relating to manganese phosphate coatings [31,32,33,34,35,36,37,38], only a few publications are available on the fundamentals of a manganese hot dip phosphatizing process applied on steel [39,40,41,42].

The phosphating process could be applied to metal substrates such as steel, cast iron, or zinc. This type of treatment process is based on the reaction between the metal surface and liquid environment, which, as a result, forms the solution of orthophosphoric acid (H_3_PO_4_) containing Mn, Zn, and Ni cations [27,31]. The phosphating process takes place at the solid–liquid interface and proceeds until the balance is achieved, i.e., until the coating is completely formed. Another important aspect of the phosphating process is related to a very problematic by-product, iron (III) phosphate (FePO_4_) [30], which is deposited onto the metal surface in the form of a solid coverage according to the following reactions:

Fe → Fe^2+^ + 2e^−^ (1)

Fe^2+^ + PO_4_^3−^ → FePO_4_↓ + e^−^ (2)

However, the utilization of iron (III) phosphate leads to a number of problems, e.g., the deposition of the phosphated metal that may cause discoloration in the coating. The phosphating is also accompanied by hydrogen generation, which should be removed in the degassing process [24]. In order to avoid the accumulation of problematic hydrogen in the cavities, phosphating of the metal is most often performed in a rotating system. Industrial experience of the author leads to the conclusion that one of the possible solutions is the use of a mobile system which works horizontally. Moreover, a temperature decrease to about 70 °C with the simultaneous increase in the concentration of manganese and the accelerator of the phosphating process could also contribute to solving the problem of hydrogen accumulation. On the basis of the available literature, it could be stated that the phosphating process includes three main stages; (i) reaction of phosphoric acid with a metal surface, (ii) rapid growth of crystals of manganese phosphate, and (iii) the formation of coatings [35,38,39].

Recently, a few types of phosphating process modifications have been proposed, such as the application of certain additives or parameter changes during selected stages of the process. One of the proposed modifications involves the addition of accelerators, such as nitrates (V) [12,15,30], sodium dodecyl sulfate [13], nitrates (III) [17], chlorates [18], or guanidine compounds [38]. Another well-known approach requires the introduction of organic compounds, i.e., benzotriazoles [9] or tolytriazoles, leading to the reduction of the crystals’ diameter (ca 4.5 ± 2 µm) until an increased surface is finally obtained [39]. A number of other modifications that concern the replacement of the nickel cation [25,31] by copper [36] or molybdenum compounds are considered as an alternative [35]. Moreover, the addition of a zirconium compound such as zirconium hexafluoride acid (H_2_ZrF_6_) [43], a titanium compound like titanium hexafluoride acid (H_2_TiF_6_) [43,44,45], or silicon compound such as silicon hexafluoride acid (H_2_SiF_6_) [35,38] to the phosphating bath, was reported to be an alternative to the traditional electrolyte composition. Such an approach allows changing of the frictional properties, corrosive behavior, abrasion resistance, tensile strength, or mechanical resistance. In addition to the manganese phosphatization processes, silicon compounds (3-triethoxysililpropylamine) [43] and urea derivatives (thiourea) are used.

**Figure 1:**
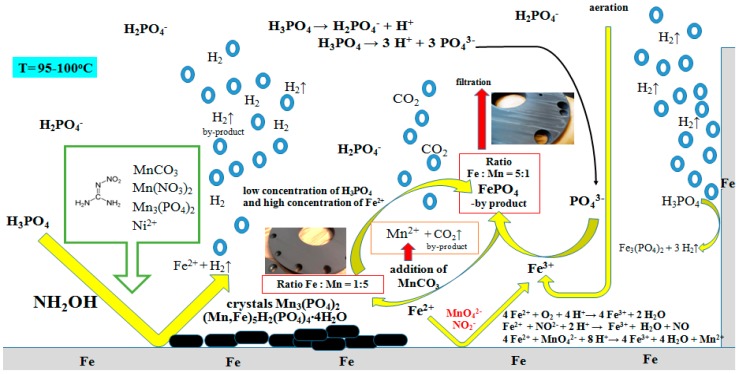
A suggested and simplified mechanism of the manganese phosphating process.

The simplified mechanism of the manganese phosphatizing coatings treatment applied to the low-alloy steel plates is presented in Figure 1. The obtained coating is affected by manufacturing conditions, such as high temperature (approximately 98 °C), the concentration of manganese, iron (II), nickel, or the content of phosphoric acid. The utilized bath contains dissolved compounds which are the source of manganese, nickel, and for the passivation process: zirconium and silicon ions.

Reactions occurring between the phosphatizing solution and metal surface are shown in Figure 1. At the beginning of the process, a dissociation of H_3_PO_4_ into H_2_PO_4_^−^ ion between pH 1 to 3 takes place (Equation (3)) [30].

H_3_PO_4_ → H_2_PO_4_^−^ + H^+^ (3)

3Mn^2+^ + 2H_2_PO_4_^−^ → Mn_3_(PO_4_)_2_ + 4H^+^ (4)

Next, phosphoric acid reacts with metal which is oxidized to ferrous cation (Fe^2+^) followed by reaction between phosphate anion and ferrous anion, resulting in the formation of ferric phosphate [30]. During this reaction, the protons from an acidic solution and are reduced to hydrogen (H_2_) [30] according to the reactions shown below:

Fe → Fe^2+^ + 2e^−^ (5)

2H^+^ + 2e^−^ → H_2_ (6)

Fe^2+^ + PO_4_^3−^ → FePO_4_ + e^−^ (7)

Finally, the manganese phosphate is precipitated (Equations (8) and (9)). The optimal pH is above 2.5 [30].

3Mn^2+^ + 2H_2_PO_4_^−^ → Mn_3_(PO_4_)_2_↓ + 4H^+^ (8)

5Mn^2+^ + 4H_2_PO_4_^−^ → Mn_2_H_2_(PO_4_)_4_ + 6H^+^ (9)

The use of a reaction accelerator prevents interaction between the metal surface and emitted bubbles of hydrogen. The presence of iron on the second oxidation rate in the bath results in the formation of a coating consisting of a mixed hureaulite, i.e., a mixed iron–manganese orthophosphate with the sum formula (Mn, Fe)_5_H_2_(PO_4_)_2_·4H_2_O.

The phosphating process needs to be modified in order to remove inappropriate color and to improve several other properties such as corrosion resistance. Therefore, in terms of protecting metals against corrosion, the optimized conditions of the phosphating process should be elaborated taking into account the desire to lower the amount of environmentally hazardous by-products and duration of each stage, which significantly affects anti-corrosion resistance of the sample as well as the cost of the treatment. The reciprocating sliding friction and wear behavior of manganese phosphate coating, deposited on the surface of mild steel substrates, was recently explored by Azhaarudeen et al. [46]. It was revealed that the wear mechanism is different in the case of uncoated mild steel and substrate coated by the manganese phosphate layer. The mechanical properties of the manganese phosphate coating were compared to those of zinc phosphate [47,48]. It was observed that zinc phosphate has a lower shear strength and wear resistance compared to manganese phosphate coating. On the basis of the available literature, it could be stated that there is a lack of data regarding a modified manganese phosphating process and the effect of Ba, Sr, Zn, Ca, Cu, Cd, and Ce addition on the passivation, activation, and phosphating processes has not been investigated yet. Another important challenge in this area is lowering the temperature of the phosphatizing process.

Bearing this in mind, we present an innovative study on the implementation of the manganese phosphate bath with compounds of manganese and nickel and the passivation bath with zirconium and silicon compounds. Another important aspect of this study is to investigate the effect of various process parameters, such as the concentration of nickel, the concentration of iron (II), or addition of accelerators on the size of crystals of manganese phosphate. In this work, a proper selection of compounds, such as guanidine derivatives e.g., 1-methyl-nitroguanidine was used to accelerate the phosphating process (urea was used to reduce the speed of the phosphating process). Moreover, the addition of a solution containing hydrogen peroxide and 1,2,3-benzotriazole to the acid at the stage of etching was proposed and investigated [9].

The effect of each process on the morphology of manganese phosphate crystals was examined by scanning electron microscopy (SEM), whereas X-ray diffraction (XRD) was used for crystal structure investigation. Energy dispersive X-ray spectroscopy (EDX) was performed for the selected samples in order to verify their composition and the uniformity of Fe, Ni, and Mn distribution. On the basis of the results of electrochemical measurements, the corrosion currents and rates were determined and finally, the optimized phosphating conditions were indicated.

## 2. Experimental

### 2.1. Samples

In this work, thin steel plates made of the low-alloy steel with dimensions of 20 mm × 20 mm and a thickness of 0.8 mm were used. To prevent corrosion of the steel, the samples were stored under a layer of chloroform. The chemical composition of these materials was analyzed with Optical Emission Spectrometer Solaris CCD Plus produced by GNR Analitycal Instruments (Nottingham, UK) and are summarized in Table 1.

The manganese phosphating coating was prepared according to the procedure described in Table 2. To determine the effect of nickel and manganese on the quality of a phosphate coating two low-alloy steel samples of the same size were prepared (2 cm × 2 cm). The amount of nickel in the phosphating bath varied from 0 to 1 g Ni(NO_3_)_2_∙6 H_2_O per 1 dm^3^, which is the equivalent of 0.0 to 0.12 g Ni per 1 dm^3^ of phosphating solution. The samples differed only in the weight of the added nickel (II) nitrate (V) added to phosphating bath. 

The set of samples has been prepared according to the conditions given in Table 2. The dose of manganese in the phosphating bath was 4 g/dm^3^ in the form of manganese carbonate (MnCO_3_). The samples differed only in the mass of manganese carbonate (MnCO_3_) added to the phosphating bath. The phosphating bath was made in accordance with the given experimental procedure.

### 2.2. The Microstructure of Manganese Coating

Complex characterization of protective manganese phosphate coatings was carried out by scanning electron microscopy SEM and XRD analysis. Quantitative analysis of the manganese coating was performed with the use of a mapping method. The morphology of fabricated materials was performed by a scanning electron microscope Quanta (FEI) equipped with an EDS accessory, enabling analysis of the elemental composition. Results have shown that the layer covering the metal plate is not consistent. There is an observable crystalline precipitate on the surface. Crystallite size varies (most of them even have a few micrometers in length). Apart from the precipitation, the crystallites are observed in the shape of a cylinder with a diameter of about 0.5–1 mm.

### 2.3. X-ray Diffraction Analysis

X-ray diffraction (XRD) was used to determine the phase composition and crystallite size. Measurements were performed on a Philips/PANalytical X’Pert Pro MPD diffractometer with a Cu K_α_ source (λ = 1.5404 Å). Data were collected from 2Θ = 5° to 70° with a scan speed of 1.1 deg/min at room temperature. The average crystallite size was estimated on the basis of the Scherrer equation taking into account reflections originating from (200) and (110) crystal phases and located at around 10–11°.

### 2.4. Electrochemical Measurements

The electrochemical measurements were performed in a standard three-electrode arrangement using a potentiostat/galvanostat, PGStat302N (Metrohm, Herisau, Switzerland). The sample of low-alloy steel after particular modification was used as a working electrode (WE) with an exposed surface area of 4 cm^2^. The reference electrode was Ag/AgCl/3MKCl (REF) and the Pt mesh was used as a counter electrode (CE). The electrochemical cell was filled with 150 mL of aerated 0.5 M KCl solution that was changed for each sample. The linear voltammetry (LV) scans were registered from the cathodic towards anodic potentials at a sweep rate of 1 mVs^−1^. The linear Tafel segments of the cathodic and anodic curves were extrapolated to the corrosion potential to obtain the corrosion current densities. The corrosion rate was calculated on the basis of the equation: CR (mpy) = (0.13·J_corr_·E.W.)/d where mpy is defined as mils per year, J_corr_ is the density of the corrosion current (µAcm^−2^), E.W. is the equivalent weight (for low-alloy steel it equals 27.9 g), and d is the density of the sample material (7.87 g cm^−3^) [43,44]. The error in the corrosion current density equals 0.01 µA cm^−2^ whereas in the case of the corrosion rate it equal to 0.0046 mpy.

This objective of these tests was to determine the effect the additives have on the corrosion resistance of the manganese phosphate coating and to determine the protective properties of the phosphate coating.

## 3. Results and Discussion

Samples were prepared using method number 2.1, as indicated in Table 2 according to the increasing numbering of stages of the process. In total, nine stages of manganese phosphating occur in the coating process. The following factors were taken into account; the effect of surface activation, the concentration of nickel in the phosphate bath, the influence of manganese on the coating quality, the effect of time on the formation of crystals of manganese phosphate, the effect of the addition of carbon manganese into the phosphating bath, and the effect of the passivation with zircon and silicon compounds. All the above processes strongly affect the creation of a uniform coating.

### 3.1. The Impact of Time of Phosphating Process

**Figure 2:**
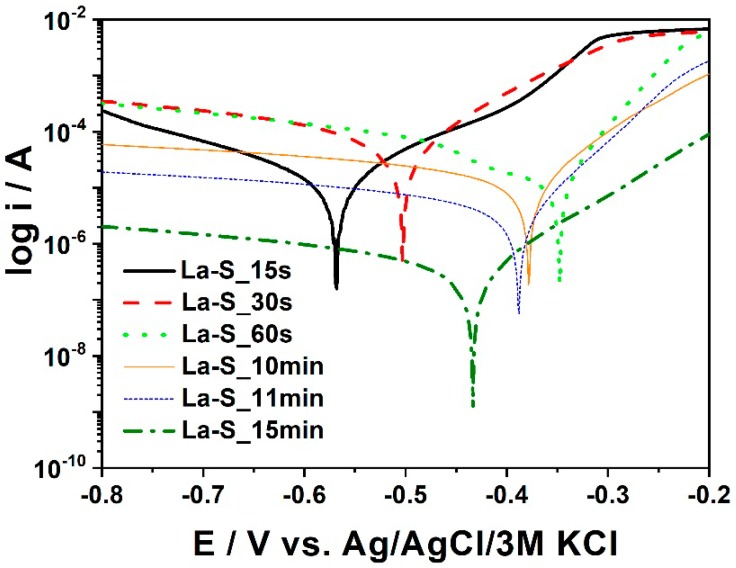
Polarization curves for selected La-S samples.

**Figure 3:**
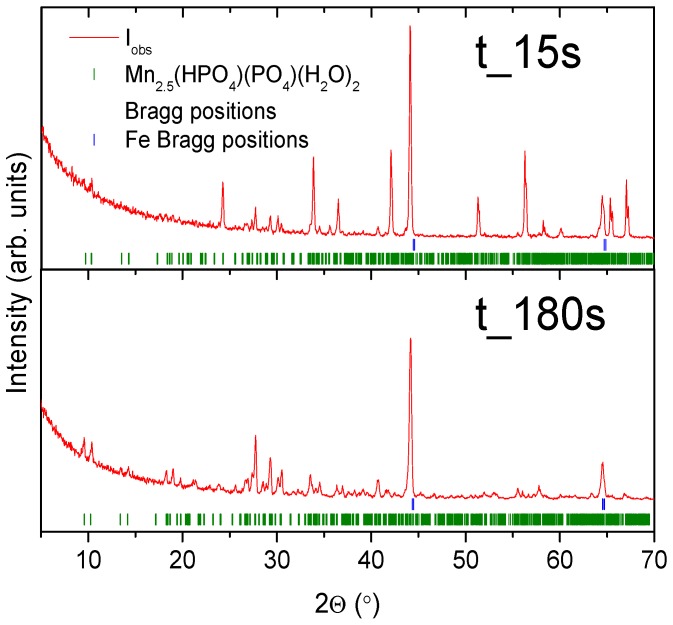
XRD patterns for the samples t_15s and t_180s. Solid red lines are the measured patterns, the green tickmarks correspond to the Mn_2.5_(HPO_4_)(PO_4_)(H_2_O)_2_ phase, and the blue tickmarks correspond to iron.

The duration of the phosphating process plays a significant role. Over time, the formation of manganese phosphate crystals increases. This paper investigates the effect that phosphating time has on the formation of the previously mentioned crystals for 14 samples with a time range from 15 s to 900 s, see the sixth stage from Table 2. A total of 14 doubled samples were prepared in the same way depending on the time of immersion in the phosphate bath and were subjected to SEM and XRD tests as well as electrochemical tests. On the surface of a sample submerged for a shorter time (15 s), several phases, including Mn_2.5_(HPO_4_)(PO_4_)(H_2_O)_2_, were detected. The observed phases are purely crystallized and hence the obtained XRD pattern is of low quality and no quantitative analysis is possible. The lower pattern presents XRD data for a sample submerged for a longer time (180 s) and shows that the sample is coated by Mn_2.5_(HPO_4_)(PO_4_)(H_2_O)_2_ phase with an estimated crystallite size of approximately 50 nm. The samples differed only with the mass of the phosphate coating. For sample La-S_15s to La-S_900s the phosphating bath was made in accordance with Table 2. The polarization curves obtained for the samples immersed for different times in the bath containing phosphate are shown in Figure 2. On the basis of the registered curves, the values of corrosion potential, current density, and corrosion rates were determined and listed in Table 3. Figure 3 presents XRD patterns for two selected samples that were phosphated for various times.

In industrial technology, time is of great importance. Some studies, such as the study by Reyes, Fuentes-Aceituno, and Salinas-Rodriguez [1], indicate a 60 min residence time in a bath. Industrial production during which the phosphated metal stays in the bath for 60 min is insufficient. The solution with the use of accelerators shortens this time from 60 min to 15 min.

In the first minutes of the phosphating process, iron oxide and iron phosphate appear on the coating. Obtaining a phosphate coating requires the use of a high temperature, which must be kept within the range of ca. 70–90 °C [26]. At lower temperatures, the coating is characterized by low corrosion resistance. If a phosphate coating is obtained, the optimum phosphating reaction time is 12–15 min [26].

**Figure 4:**
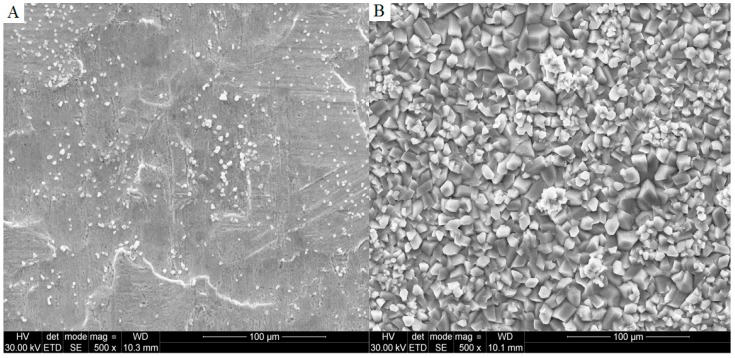
SEM images of as-prepared samples: (**A**) After 15 s; and (**B**) after 900 s of the process.

It could be observed that the samples which are labeled as La-S_60s–L-S_180s had the highest (less negative) corrosion potential among the materials. The most negative values were reached for the samples immersed for 15, 30, and 540 s. Following the analysis of the results for the samples immersed in a phosphate bath for a longer time, the dependence of the corrosion rate of two samples, La-S_15s and La-S_30s, in this group is not taken into account because the processing time was too short. It proves that the limited immersion time results in the corrosion process taking place on the metal surface rather than in the forming of the protective coating. The sample, La-S_900s, exhibits the lowest corrosion current value and, therefore, is less susceptible to the corrosion. The sample immersed only for 15 s (Figure 4A) could be regarded as a pure metal and treated as a reference sample in this measurement system, whereas the sample exposed for 15 min (La-S_900s) (Figure 4B) to the plating had a corrosion rate of only 0.012 (CR/mpy). This value may result from the presence of manganese phosphate and iron phosphate crystals on the metal surface. These crystals are likely to catalyze the corrosion rate.

**Figure 5:**
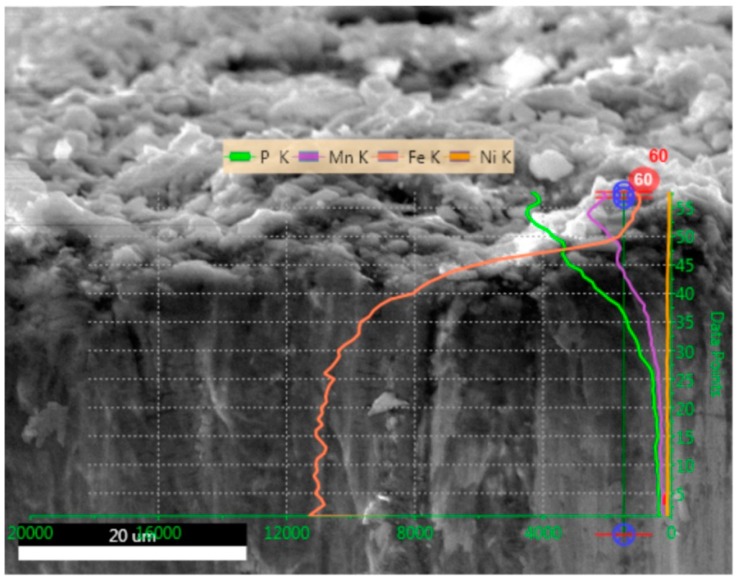
Cross-section analysis of sample B.

Figure 5 presents a cross-sectional area of sample B. A highly corrugated sample surface is seen on the top, whereas a relatively homogeneous bulk is seen on the bottom of the figure. The elemental analysis of the cross-section is represented by four solid lines that show the relative concentration of P, Mn, Fe, and Ni. On the surface P, Mn, and Fe are present and detailed analysis reveals that a relative atomic concentration is 50:37:13 (P:Mn:Fe). The concentration of Fe rapidly increases with distance from the surface. There is no clear border between the surface and a bulk material, but 15 μm inside the sample we found twice the amount of Fe compared to the total amount Mn and P together and at a distance of 30 μm from the surface with a relative atomic concentration is 2:1:97 (P:Mn:Fe). Nickel was not found in this sample.

### 3.2. The Impact of Nickel on Coating Quality

Nickel is a common addition to a phosphate bath in the phosphating process. Nickel catalyzes the formation of manganese phosphate crystals. The addition of nickel in the phosphatizing bath contributes to a tight coating with regular rhombic crystals. The most commonly used type of nickel compound is nickel (II) nitrate (V), nickel (II) phosphate (V), nickel carbonate, and the rarely used nickel (II) chloride.

In the nickel impact test, the samples were labeled Ni_0 to Ni_0.15, see details in Table 4. Each sample was synthesized twice.

**Figure 6:**
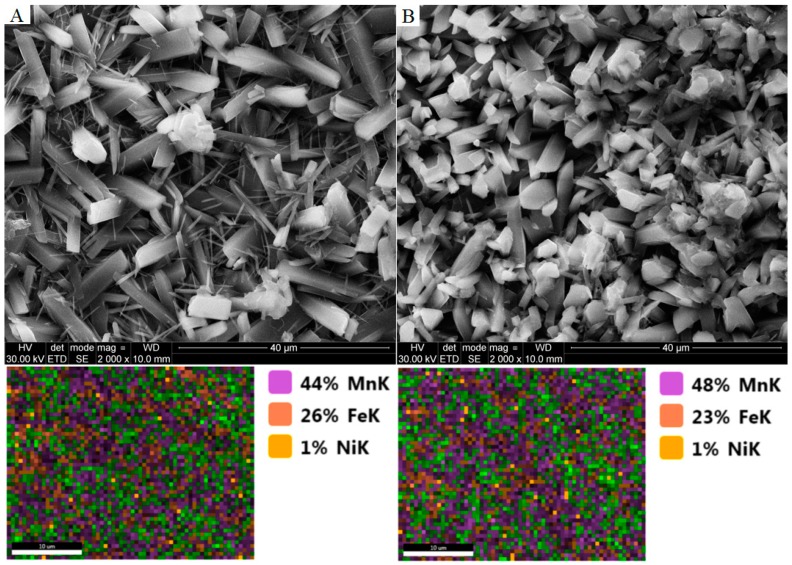
SEM images of coating: (**A**) the manganese phosphating coating without nickel; (**B**) the manganese phosphating coating containing nickel.

Based on the photos taken with the scanning microscope, differences in the structure of the crystals are evident, see Figure 6. The phosphate coating obtained with the addition of nickel is in the form of crystals with a regular rhombic structure. The coating obtained in a bath without the addition of nickel is in the form of crystals with the appearance of needles.

**Figure 7:**
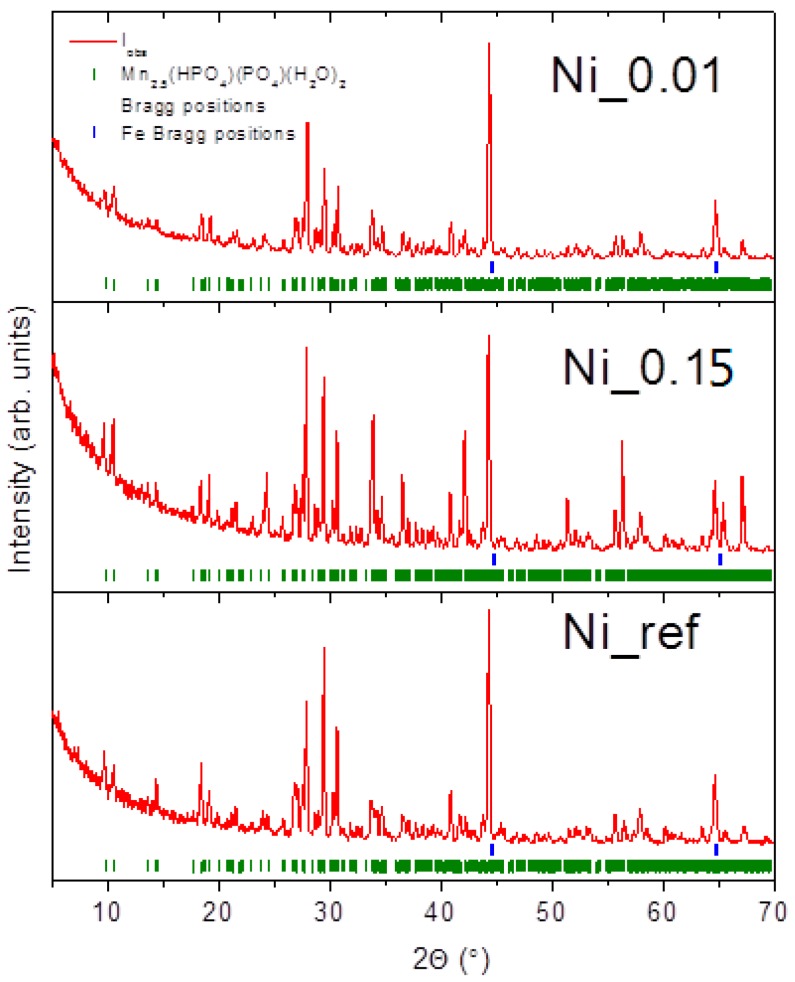
XRD patterns for the samples Ni_0.01, Ni_0.15, and Ni_ref. Solid red lines are measured patterns, the green tickmarks correspond to the Mn_2.5_(HPO_4_)(PO_4_)(H_2_O)_2_ phase, and the blue tickmarks correspond to iron.

Figure 7 shows the XRD patterns for some samples which were phosphated in baths with various amounts of nickel. It can be observed for all the samples that a coating made of Mn_2.5_(HPO_4_)(PO_4_)(H_2_O)_2_ and an iron phase were not present. Changes in intensity peaks and average crystallites sizes were noticed; however, they seem to be independent of the concentration of nickel in the baths. The average crystallites sizes were in the range of 26–64 nm.

**Figure 8:**
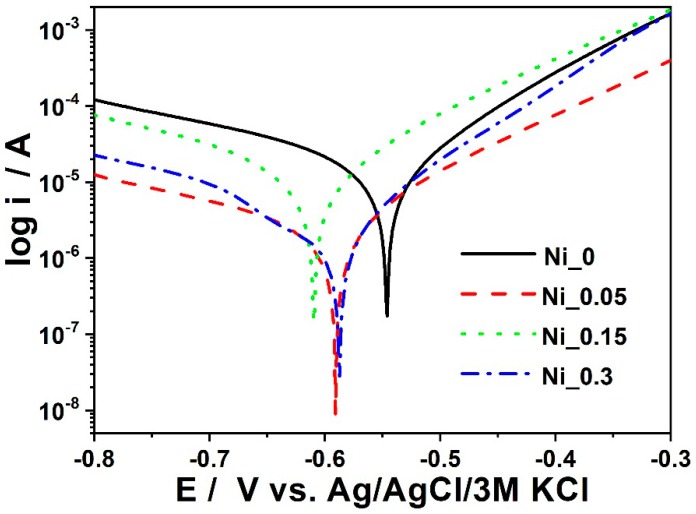
Polarization curves for samples Ni_0 to Ni_0.3.

The analysis of the results for samples Ni_0–Ni_0.3 listed in Table 5 proves that the sample Ni_0.3 shows the lowest corrosion rate. The smallest corrosion rate was determined for Ni_0.05 and Ni_0.3 but the Ni_0.05 sample has worse anti-corrosive properties than the Ni_0.3 sample. The sample Ni_0.3 has the best anticorrosion properties. According to the collected data, the corrosion resistance (Figure 8) of the coating increases with the increasing concentration of nickel in the bath (Table 6). As a result, the concentration of nickel could be regarded as a factor which significantly influences the corrosion rate of the obtained material. Taking into account the morphology, it affects the number of micro cathodes on the metal surface, thus increasing the number of active sites of the phosphate crystal.

### 3.3. The Impact of Manganese on Coating Quality

**Figure 9:**
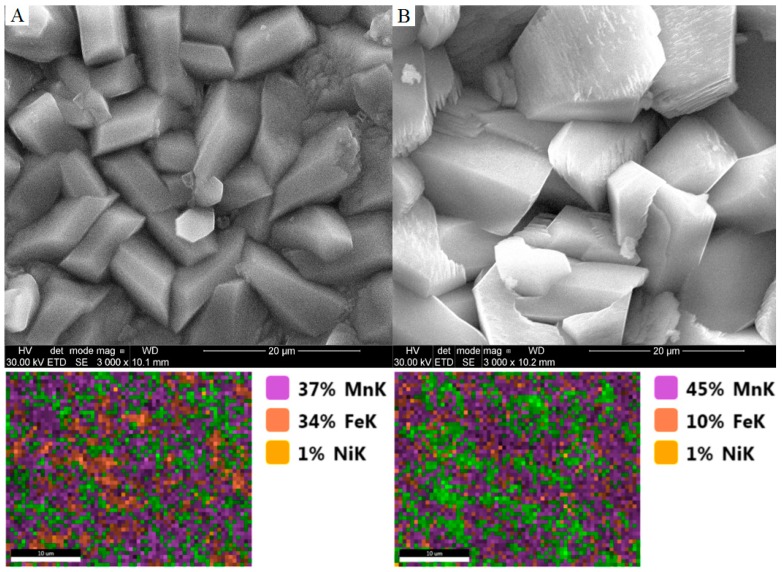
SEM images and EDX analysis of obtained coatings: (**A**) The manganese phosphating coating without the addition of manganese carbonate; (**B**) the manganese phosphating coating with the addition of manganese carbonate.

Manganese is the basic building block of the phosphate manganese conversion coating. This part of the work examines the effect of changing the concentration of manganese in a phosphate bath on the nature of the obtained coating. Two samples have been prepared in accordance with Table 2. Manganese in a phosphate bath occurs in the form of Mn^2+^ cations. In order to increase the concentration of manganese in phosphate baths in industrial production, compounds such as manganese (V) nitrate, manganese (V) phosphate (II), or manganese carbonate are used. In the manganese phosphatizing process, the use of manganese compounds in which the manganese is in the +7 oxidation state, Mn^7+^ (permanganate), is most often used to remove iron phosphate from the bath. Iron (II) ions are then oxidized to iron (III) ions. Based on the photos taken with the scanning microscope, you can see differences in the structure of the crystals. The high concentration of manganese in the phosphatizing bath results in crystals with characteristic features and cracks (Table 7). Manganese phosphate crystals that build a coating obtained from a bath without the addition of manganese carbonate will have rounded edges, as shown in Figure 9.

**Figure 10:**
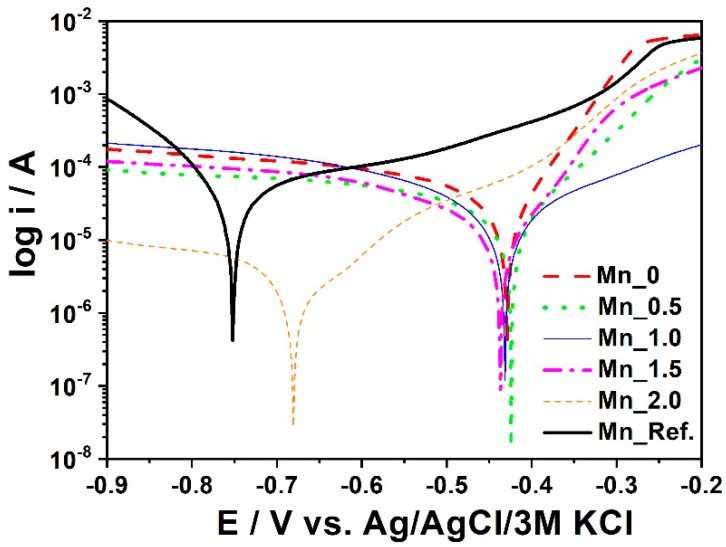
Polarization curves registered for samples with different Mn contents.

**Figure 11:**
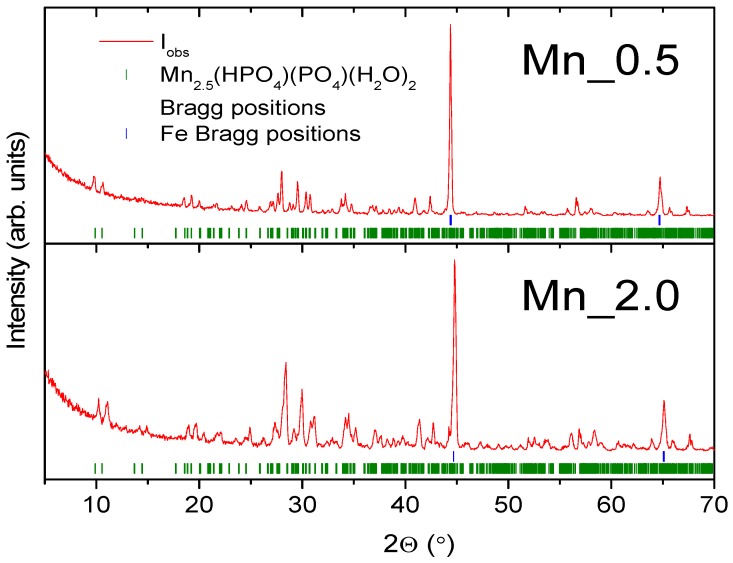
XRD patterns for the samples Mn_0.5 and Mn_2.0. Solid red lines are measured patterns, the green tickmarks correspond to Mn_2.5_(HPO_4_)(PO_4_)(H_2_O)_2_ phase, and the blue tickmarks correspond to iron.

Figure 9 presents SEM images and Figure 10 shows polarization curves registered for Mn_0 to Mn_2.0. A reference sample was also used. XRD patterns for two phosphated samples with different Mn contents in the bath are shown in Figure 11.

In this study, the manganese concentration in the phosphate bath was increased by using manganese carbonate, which readily reacts with the phosphoric acid contained in the bath to form the (V) manganese (II) phosphate according to the following reaction:

3MnCO_3_ + 2H_3_PO_4_ → Mn_3_(PO_4_)_2_ + 3CO_2_↑ + 3H_2_O (10)

The results of electrochemical measurements are presented in Table 8.

It was noticed that with the increase of the manganese concentration in the phosphatizing bath, smaller crystals were obtained, which improves the tightness of the coating and thus the corrosion resistance, as confirmed by electrochemical tests.

Among all manganese-modified materials, the Mn_2.0 sample shows the highest corrosion potential and a relatively low corrosion current. It demonstrates the best anti-corrosive properties. It could also be noticed that the reference sample is characterized by the worst electrochemical activity towards corrosion protection compared to other materials. With the increase of manganese concentration in the phosphating bath, the anti-corrosion properties of the manganese phosphate coating are improved.

In both cases, strong diffraction peaks attributed to the iron plate and Mn_2.5_(HPO_4_)(PO_4_)(H_2_O)_2_ coating are observed. The relative intensity of the manganese hydrogen phosphate hydrate phase increases with growing manganese content in the phosphating bath. A low-alloy-steel sample of the same size as the Mn_2.5_(HPO_4_)(PO_4_)(H_2_O)_2_ phase is the smallest for the manganese Mn 2% bath with d~32 nm, whereas, for the lowest Mn concentration bath, the crystallite size is approximately 36 nm.

### 3.4. The Impact of Activation and Passivation on Coating Quality

**Figure 12:**
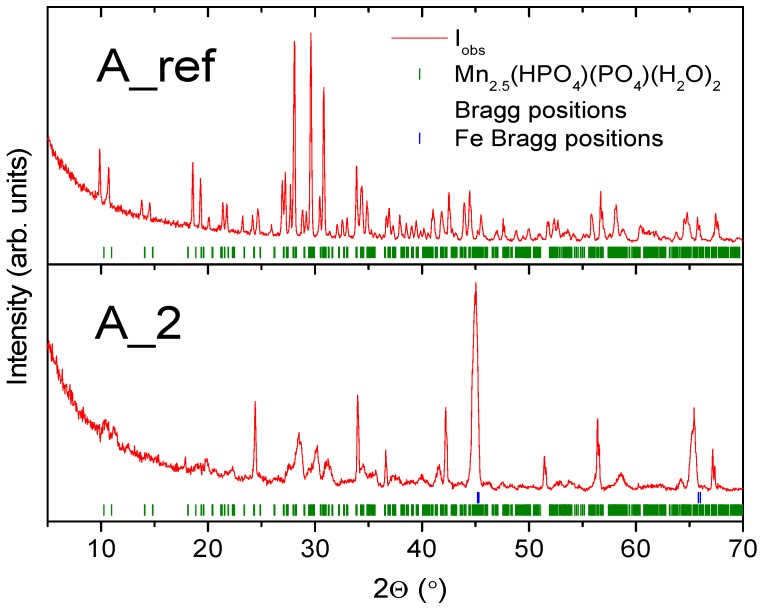
XRD patterns for the A_ref and A_2 samples. Solid red lines are measured patterns, the green tickmarks correspond to Mn_2.5_(HPO_4_)(PO_4_)(H_2_O)_2_ phase, and the blue tickmarks correspond to iron.

**Figure 13:**
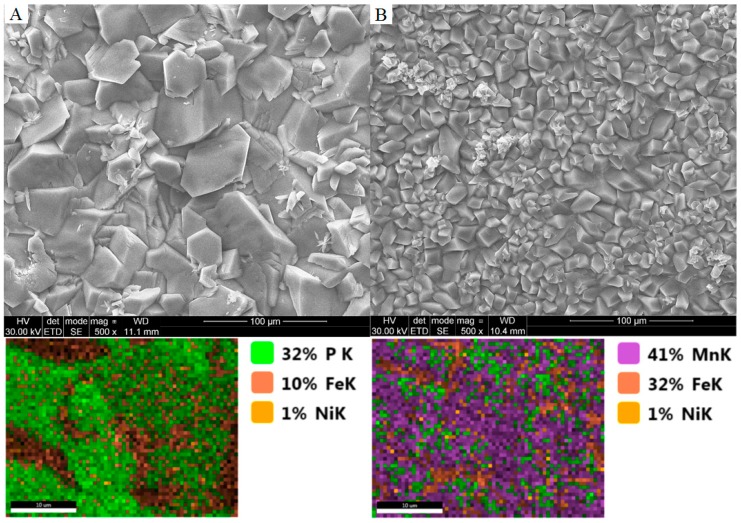
SEM images of the obtained coatings: (**A**) The manganese phosphating coating without the activation process; and (**B**) the manganese phosphating coating with the activation process.

In order to determine the effect of the activation process on the quality of a manganese phosphate coating, five samples of the low-alloy steel were prepared. Each sample was phosphated according to Table 2, and the samples prepared in individual conditions were doubled. The XRD patterns for a reference and selected activated samples are shown in Figure 12. The coating after the activation process in an alkaline dilution and is characterized by good tightness. The size of the crystals varies between 2 and 10 μm. The coating obtained in the phosphating process without activation is formed with large crystals. Their size is within the range of 50–60 μm, see Figure 13.

Results show that the coating of the reference sample is made of a Mn_2.5_(HPO_4_)(PO_4_)(H_2_O)_2_ (manganese hydrogen phosphate hydrate) phase, with an average crystallite size of 100 nm. In the case of A_2, see Figure 13B, the sample coating was made of two phases; the same as observed for a reference sample and Fe_2_PO_4_(OH). The low quality of the XRD pattern is due to low crystallinity and precludes crystallite size estimation. 

As it was shown in the sample groups discussed above, the typical peaks for the iron substrate and manganese hydrogen phosphate hydrate phase are observed. In this case, no clear dependence between passivation time and average crystallites size was observed. The average crystallites sizes are in the range of 23–32 nm.

As can be seen in Figure 13, after 15 min of immersing the sample in a phosphate bath, depending on the sample activation, different crystal sizes are obtained. The manganese phosphate coating obtained without the activation process is visible to the naked eye. However, the coating is unstable due to the size of the crystals as it is destroyed more quickly due to the fragility of the coating.

Samples were subjected to the activation process in a bath containing manganese hydrogen phosphate and a balanced amount of sodium pyrophosphate. The results of the SEM analysis are presented in Table 9. And the results of the analysis of electrochemical measurements are presented in Table 10.

**Figure 14:**
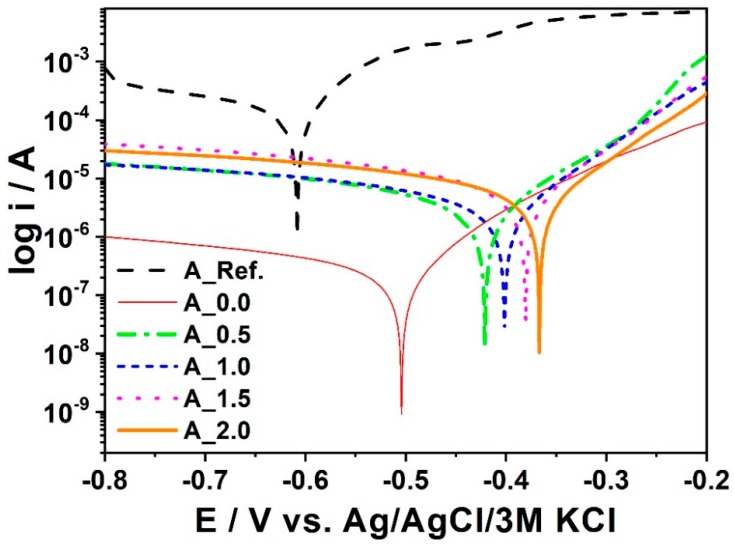
Polarization curves registered for samples subjected to the activation process.

Comparing the values collected in Table 10, the sample A_2.0 exhibits the best anti-corrosion properties such as the highest corrosion potential and a relatively low corrosion current. The results of electrochemical measurements, see Figure 14, indicate that the activation of the metal surface has a high impact on the corrosion rate of the phosphate coating. It has been shown that the concentration of the activating bath has a significant effect on the corrosion resistance. Better results were obtained at a temperature of 45 °C. The best anti-corrosion properties were obtained when samples were treated in a bath containing 2 g of MnHPO_4_ and Na_4_P_2_O_7_ per 0.5 L water.

**Figure 15:**
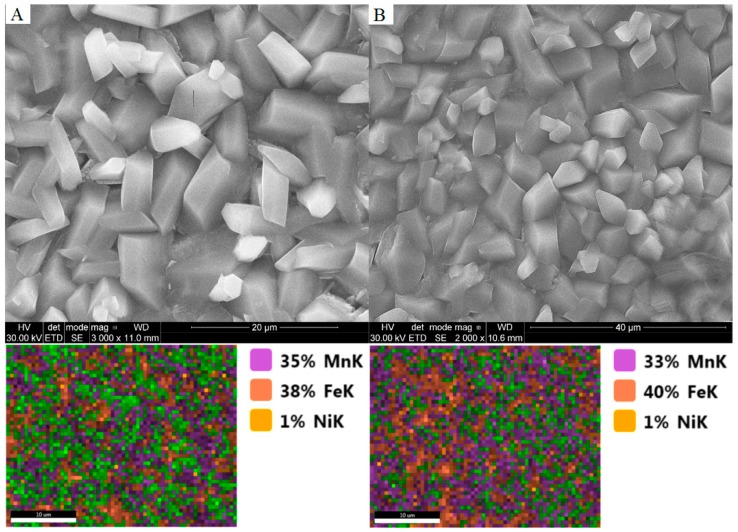
SEM images and EDX analysis of obtained coatings: (**A**) Without the passivation process; and (**B**) after the passivation process.

To determine the effect of the passivation process on the quality of a phosphate coating two low-alloy steel samples of the same size were prepared, see Figure 15. One sample was prepared with the passivation and oiliness process and another sample was prepared with the passivation process only. In the oiliness process, a solution of mineral oil, emulsifying with water (ZWEZ E-4999) was used. The passivating process of the samples was performed in a bath containing zirconium and silicon compounds. 

**Figure 16:**
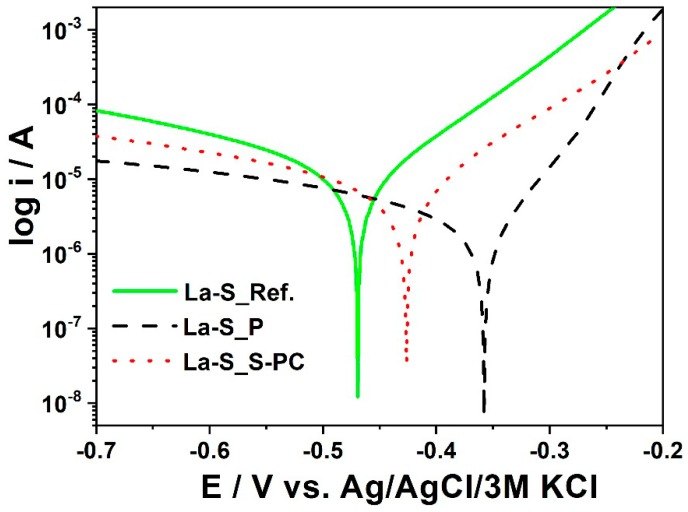
Polarization curves obtained for samples La-S_Ref, La-S_P, and La-S_PC.

**Figure 17:**
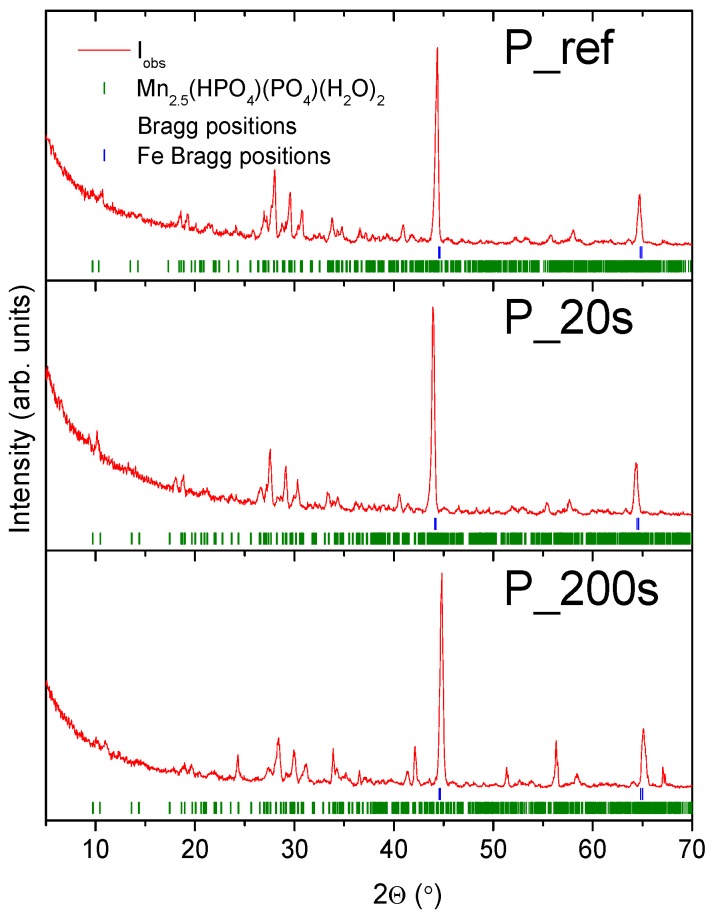
XRD patterns for the P_Ref, P_20s, and P_200s samples. Solid red lines are measured patterns, the green tickmarks correspond to Mn_2.5_(HPO_4_)(PO_4_)(H_2_O)_2_ phase, and the blue tickmarks correspond to iron.

The passivating bath was made in accordance with information given in Table 11. In Figure 16 polarization curves were registered for samples subjected to a passivation process. To determine the effect of an oiliness process on the quality of a phosphate coating, two samples of the same size of low-alloy steel were prepared. Each sample was prepared according to Table 2. Two strong Bragg reflections of iron originating from the substrate were also observed. XRD patterns for the samples which were passivated for different times are presented in Figure 17.

It could be observed that the La-S_P sample has the highest corrosion potential and a relatively low corrosion current. Therefore, this material is considered as having the best anti-corrosion properties. These studies revealed that the conservation process with the use of the mineral oil together with the passivation treatment accelerates the corrosion progress. However, the use of passivation without mineral oils increases the corrosion resistance of the sample with a manganese phosphate coating, as is observed in Figure 16. The sample denoted as La-S_P has the lowest value of corrosion rate and the smallest corrosion current density, see Figure 16.

Another type of passivation process was the process with ZrOCl_2_ and H_2_SiF_6_. The materials labeled Si_Ref and Zr_Ref were fabricated without the use of Si and Zr. The C_Ref sample was not conserved in the mineral oil, whereas the C_Oil sample was immersed in the mineral oil. The sample containing zirconium was immersed in a bath containing ZrOCl_2_ according to the description given in Table 2.

**Figure 18:**
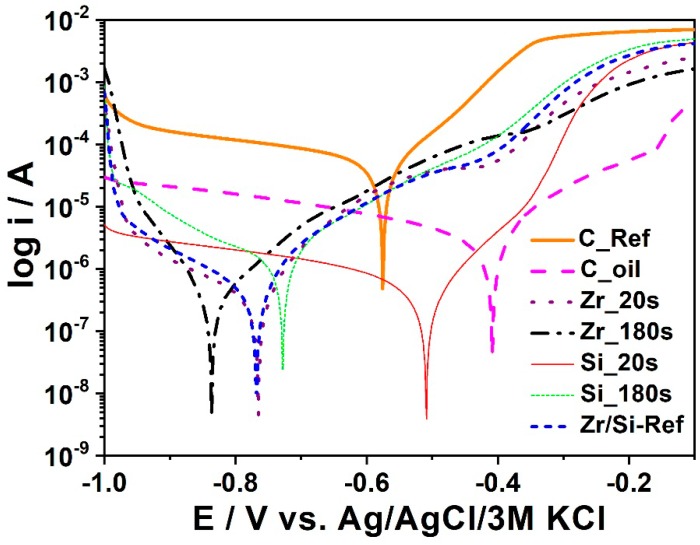
Polarization curves obtained of the Zr_20s, Si_20s, Zr_180s, Si_180s, Si_Ref, Zr_Ref, C_Ref, and C_Oil samples.

Among all the samples subjected to the passivation and the conservation process with mineral oil, the Zr_180s sample, see Figure 18, exhibits the highest corrosion potential and relatively low corrosion current and it was subsequently identified as the best-protected sample against corrosion.

### 3.5. The Impact of Etching on Coating Quality

In galvanic processes, one of the basic elements is the removal of rust deposits. Removal of rust and scale is important when creating a uniform coating. Rust and scale are not removed at the degreasing stage; hence, the use of acids is required. The most commonly used acids are sulfuric acid (VI), phosphoric acid, or hydrochloric acid. Due to the high costs of sulfuric acid (VI) and phosphoric acid, the most preferred is the use of hydrochloric acid. In order to accelerate the process of removing rust and scale deposits, the addition of hydrogen peroxide was used in this study. Two samples for each condition have been prepared, see Table 12.

During the phosphating process, samples are exposed to acids such as hydrochloric, sulfuric, and phosphoric acid. The acidity level of the phosphating solution was the same (total acidity was 82 point, free acidity was 14.2 points, and the concentration of iron equaled 0.42 g/L). In order to determine the effect of the acid, various combinations were used with sulfuric acid, phosphoric acid, and hydrochloric acid. The samples were labeled with the use of Et_H_3_PO_4,_ Et_HCl-Ni, Et_H_2_SO_4_-Ni, and Et_H_3_PO_4_-Ni, see Table 12.

**Figure 19:**
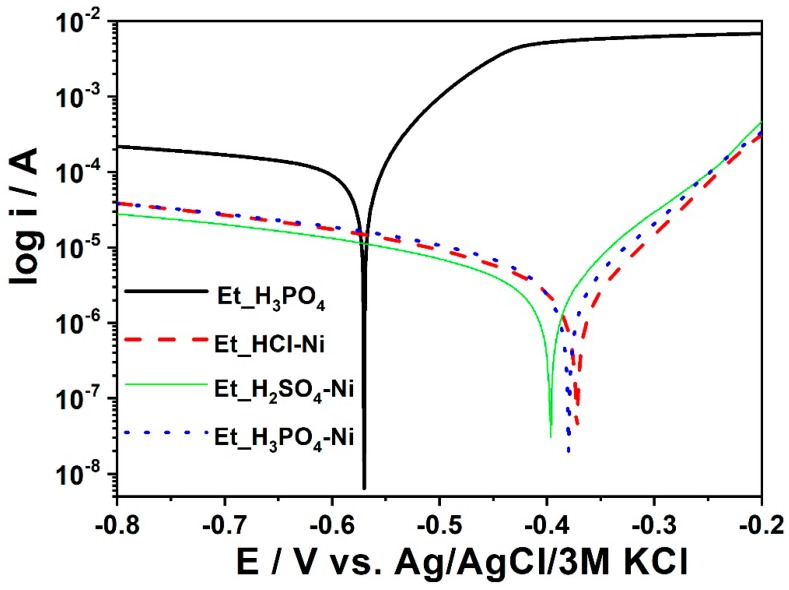
Polarization curves obtained for the Et_H_3_PO_4_, Et_HCl-Ni, Et_H_2_SO_4_-Ni, and Et_H_3_PO_4_-Ni samples.

According to the results collected for the series of materials labeled as Et_H_3_PO_4_, Et_HCl-Ni, Et_H_2_SO_4_-Ni, and Et_H_3_PO_4_-Ni, see Figure 19, the sample Et_H_3_PO_4_-Ni exhibits the best anti-corrosive properties; that is, a less negative corrosion potential and quite a low corrosion rate.

## 4. Conclusions

The following conclusions are formed on the basis of this study:

(1) The addition of manganese in the form of manganese carbonate dramatically improves the phosphate coating. Similarly, the addition of nickel dramatically affects the quality of the phosphate coating achieved.
(2) The activation of the surface of a metal sample just before the phosphating process begins significantly affects the corrosion resistance. Along with an increase in the concentration of manganese hydrogen phosphate and sodium pyrophosphate, smaller crystals are formed, which improves the tightness of the coating.
(3) It has been shown that the passivation process with the use of hexafluorozirconium acid or zirconyl chloride and hexafluorosilocon acid does not increase the concentration of zirconia and silicon in the manganese phosphate coating. The usage of zirconia chloride leads to good corrosion resistance.
(4) The addition of manganese and nickel to the phosphating bath reduces the rate of corrosion of the sample. Also, increasing the concentration of the activating bath dramatically reduces the corrosion rate of the sample. It has been discovered that a higher concentration of nickel in the phosphating bath increases the anticorrosion properties of the coating. During the analysis, the optimal dose of the nickel (II) nitrate (V) was determined to be 0.6 g/1 dm^3^. The process without the use of nickel results in a coating which has crystals of different shapes.
(5) With the increasing concentration of manganese, and without changing the acidity and the free acidity, the corrosion resistance of the phosphate coating increases. During the polarization measurements it can be seen that with an increasing formation time of a phosphate coating, the anticorrosive properties increase.
(6) XRD analysis indicated the presence of two phases in the phosphate coating; Fe metal phase and phosphate coating phase, Mn_2.5_(HPO_4_)(PO_4_)(H_2_O)_2_. The cross-section analysis showed that the manganese coating had no clear border between the surface and the metal. A relative atomic concentration on the surface is 50:37:13 (P:Mn:Fe) and 30 μm from the surface is 2:1:97 (P:Mn:Fe).
(7) On the basis of electrochemical tests, it has been proven that with an increased immersion time of the sample in the phosphatizing bath, the corrosion resistance increases. The best effect was obtained for a sample with a time of 900 s in a phosphate bath.
(8) The use of phosphoric acid in the process of sample etching results in a higher corrosion resistance of the coating. Furthermore, the addition of hydrogen peroxide to the etching bath affects the phosphating coating conditions.

## Figures and Tables

**Table 1:** The detailed chemical composition of the low-alloy steel.

Element	**C**	**Si**	**Mn**	**P**	**S**	**Cr**	**Mo**	**Ni**	**Nb**	**Al**	**Cu**
Weight content (%)	0.034	0.009	0.220	0.009	0.007	0.040	0.007	0.012	0.011	0.026	0.041
Element	**Co**	**B**	**Ti**	**W**	**Ca**	**Sn**	**Pb**	**Sb**	**Te**	**As**	**Fe**
Weight content (%)	0.002	<0.0001	0.010	0.008	0.001	0.001	0.006	0.008	0.001	0.001	99.545

**Table 2:** The chemical composition of manganese phosphating treatment and process parameters.

Stage No	Process Type	Composition of Solution	Process Conditions
**1**	Degreasing	10% (or 7 mol) solution of NaOH	Mixing, 80–85 °C, 5 min
**2**	Rinsing	Distilled water	room temp., 3 min
**3**	Etching	Solution of HCl 15%, 1–10 mL of 3% H_2_O_2_	room temp., 3 min
**4**	Rinsing	Distilled water	room temp., 3 min
**5**	Activating	MnHPO_4_: 2 g	mixing, 40–45 °C, 4 min
		Na_4_P_2_O_7_: 2 g	
		H_2_O: 500 g	
**6**	Phosphating	H_3_PO_4_ (85%): 7.0 g	95 °C, 15 min
		Mn_3_(PO_4_)_2_: 15.0 g	
		Mn(NO_3_)_2_: 6.0 g	
		MnCO_3_: 0.5 g	
		Ni(NO_3_)_2_: 0.3 g	
		H_2_O: 531.5 g	
		(optional) 1-methyl-nitroguanidine: 0.5 g	
**7**	Rinsing	Distilled water	room temp., 3 min
**8**	Passivation	ZrOCl_2_/H_2_SiF_6_: 0.3 g	Mixing, 25–30 °C, 2 min
		Mn(NO_3_)_2_: 1.1 g	
		NaNO_3_: 0.14 g	
		HNO_3_: 0.14 g	
		CH_3_OH: 0.14 g	
		Na_2_CO_3_: 5.4 g	
		H_2_O: 100 g	
**9**	Conservation with oil	Solution of mineral oil solution of mineral, emulsifying with water oil (ZWEZ 4999) produced by ZWEZ.	Mixing, 75 °C, 2 min

**Table 3:** The values of corrosion potential, current density, and corrosion rate estimated for La-S samples treated for various times in a phosphate bath.

Sample	Time of Immersion in Phosphate Bath (s)	Corrosion Potential (Ecor/V)	Current Density (jcor/µAcm^−2^)	Corrosion Rate (CR/mpy)
La-S_15s	15	−0.568	1.200	0.554
La-S_30s	30	−0.500	3.7225	1.719
La-S_60s	60	−0.347	2.2250	1.038
La-S_120s	120	−0.372	0.9975	0.460
La-S_180s	180	−0.354	0.705	0.325
La-S_240s	240	−0.396	1.2080	0.557
La-S_300s	300	−0.363	0.730	0.337
La-S_360s	360	−0.358	0.830	0.383
La-S_420s	420	−0.376	1.075	0.496
La-S_480s	480	−0.400	1.300	0.6
La-S_540s	540	−0.440	2.050	0.946
La-S_600s	600	−0.379	1.205	0.556
La-S_660s	660	−0.387	0.3925	0.181
La-S_900s	900	−0.431	0.025	0.012

**Table 4:** Sample labeling and nickel content in the samples.

Sample	Nickel Content (g)
Ni_0	0.0
Ni_0.01	0.002
Ni_0.05	0.010
Ni_0.12	0.025
Ni_0.15	0.030

**Table 5:** The values of corrosion potential, current density and corrosion rate estimated for nickel-based samples.

Sample	Nickel Content (g)	Corrosion Potential (Ecor/V)	Current Density (jcor/µAcm^−2^)	Corrosion Rate (CR/mpy)
**Ni_0**	0.00	−0.546	0.9325	0.430
**Ni_0.05**	0.01	−0.590	0.2450	0.113
**Ni_0.15**	0.03	−0.609	0.9090	0.419
**Ni_0.3**	0.06	−0.587	0.1450	0.067

**Table 6:** The chemical composition of the A (Ni_Ref) and B (Ni_0.15) samples based on EDX analysis.

Element	SEM Image A		SEM Image B	
	Weight (%)	Atomic (%)	Weight (%)	Atomic (%)
P K	30.68	44.08	32.69	46.37
Mn K	52.8	42.77	52.37	41.88
Fe K	16.24	12.94	14.57	11.46
Ni K	0.28	0.21	0.38	0.29

**Table 7:** The chemical composition for samples A (Mn_0) and B (Mn_2.0) based on EDX analysis.

Element	SEM Image A		SEM Image B	
	Weight (%)	Atomic (%)	Weight (%)	Atomic (%)
P K	20.94	32.2	17.88	14.58
Mn K	28.05	24.32	34.67	15.94
Fe K	50.48	43.05	4.65	2.1
Ni K	0.53	0.43	0.16	0.07

**Table 8:** The values of corrosion potential, current density, and corrosion rate estimated for the samples with Mn content.

Sample	Additional Manganese Content (g)	Corrosion Potential (Ecor/V)	Current Density (jcor/µAcm^−2^)	Corrosion Rate (CR/mpy)
Mn_0	0.0	−0.427	3.5000	1.615
Mn_0.5	0.24	−0.424	1.8750	0.865
Mn_1.0	0.48	−0.430	1.8890	0.871
Mn_1.5	0.72	−0.680	1.2880	0.594
Mn_2.0	0.96	−0.680	0.2470	0.114
Mn_Ref.	Reference sample	−0.750	5.2450	2.420

**Table 9:** The chemical composition for samples A (Mn_0), B (Mn_2.0), C and D based on EDX analysis.

Element	SEM Image A		SEM Image B		SEM Image C		SEM Image D	
	Weight (%)	Atomic (%)	Weight (%)	Atomic (%)	Weight (%)	Atomic (%)	Weight (%)	Atomic (%)
Na K	1.45	2.75	-	-	-	-	-	-
P K	31.26	43.97	24.81	37.1	24.12	36.26	24.45	36.7
Mn K	61.63	48.87	40.82	34.41	34.52	29.27	32.54	27.53
Fe K	5.59	4.36	33.79	28.02	40.73	33.97	42.48	35.36
Ni K	0.05	0.05	0.58	0.46	0.63	0.5	0.53	0.42

**Table 10:** The values of corrosion potential, current density, and corrosion rate estimated for samples that underwent the activation and passivation processes.

Activation Process					Passivation Process				
Sample	Activation Dose MnHPO_4_/Na_4_P_2_O_7_ (g)	Corrosion Potential (Ecor/V)	Current Density (jcor/µAcm^−2^)	Corrosion Rate	Sample	Conditions of Passivation (g)	Corrosion Potential (Ecor/A)	Current Density (jcor/µAcm^−2^)	Corrosion Rate (CR/mpy)
A_Ref	Reference sample	−0.608	11.50	5.306	La-S_Ref	ZrOCl_2_/H_2_SiF_6_: 0.3 g Mn(NO_3_)_2_: 1.1 g NaNO_3_: 0.14 g HNO_3_: 0.14 g CH_3_OH: 0.14 g Na_2_CO_3_: 5.4 g H_2_O: 100 g 25–30 °C, 2 min	−0.470	0.6575	0.303
A_0.0	0.0	−0.504	0.0195	0.009					
A_0.5	0.5	−0.420	0.2825	0.130	La-S_P		−0.357	0.1688	0.078
A_1.0	1.00	−0.401	0.2318	0.107					
A_1.5	1.5	−0.379	0.301	0.139	La-S_PC		−0.426	0.49	0.266
A_2.0	2.0	−0.366	0.293	0.135					

**Table 11:** The values of corrosion potential, current density, and corrosion rate estimated for samples chemically treated in passivation conditions.

Sample	Passivation Conditions	Process Time (s)	Corrosion Potential (Ecor/V)	Current Density (jcor/µAcm^−2^)	Corrosion Rate (CR/mpy)
Zr_20s.	ZrOCl_2/_H_2_SiF_6_: 0.3 g Mn(NO_3_)_2_: 1.1 g NaNO_3_: 0.14 g HNO_3_: 0.14 g CH_3_OH: 0.14 g Na_2_CO_3_: 5.4 g H_2_O: 100 g 25–30 °C, 2 min	20	−0.768	0.04468	0.021
Si_20s.		20	−0.507	0.04283	0.020
Zr_180s.		180	−0.836	0.0930	0.011
Si_180s.		180	−0.728	0.1174	0.054
Zr/Si_Ref	Without	-	−0.769	0.04985	0.023
C_Ref	Without	-	−0.574	4.3080	1.987
C_Oil	With oilness process	120	−0.408	0.3290	0.152

**Table 12:** The values of corrosion potential, current density, and corrosion rate estimated for samples treated in the phosphating bath containing nickel species.

Sample	Etching Conditions (%)	Nickel Content in the Phosphating Bath (g)	Corrosion Potential (Ecor/V)	Current Density (jcor/µAcm^−2^)	Corrosion Rate (CR/mpy)
Et_H_3_PO_4_	15	0.0	−0.569	7.7830	3.591
Et_HCl-Ni	15	0.3	−0.372	0.2843	0.131
Et_H_2_SO_4_-Ni	15	0.3	−0.394	0.2578	0.119
Et_H_3_PO_4_-Ni	15	0.3	−0.377	0.2978	0.137
